# Chromosome-level genome assembly and annotation of eel goby (*Odontamblyopus rebecca*)

**DOI:** 10.1038/s41597-024-02997-8

**Published:** 2024-02-02

**Authors:** Zhenming Lü, Ziwei Yu, Wenkai Luo, Tianwei Liu, Yuzheng Wang, Yantang Liu, Jing Liu, Bingjian Liu, Li Gong, Liqin Liu, Yongxin Li

**Affiliations:** 1 National Engineering Laboratory of Marine Germplasm Resources Exploration and Utilization, College of Marine Sciences and Technology, Zhejiang Ocean University, Zhoushan, 316022 China; 2 School of Ecology and Environment, Northwestern Polytechnical University, Xi’an, 710072 China

**Keywords:** Genome, Genomics

## Abstract

The eel gobies fascinate researchers with many important features, including its unique body structure, benthic lifestyle, and degenerated eyes. However, genome assembly and exploration of the unique genomic composition of the eel gobies are still in their infancy. This has severely limited research progress on gobies. In this study, multi-platform sequencing data were generated and used to assemble and annotate the genome of *O. rebecca* at the chromosome-level. The assembled genome size of *O. rebecca* is 918.57 Mbp, which is similar to the estimated genome size (903.03 Mbp) using 17-mer. The scaffold N50 is 41.67 Mbp, and 23 chromosomes were assembled using Hi-C technology with a mounting rate of 99.96%. Genome annotation indicates that 53.29% of the genome is repetitive sequences, and 22,999 protein-coding genes are predicted, of which 21,855 have functional annotations. The chromosome-level genome of *O. rebecca* will not only provide important genomic resources for comparative genomic studies of gobies, but also expand our knowledge of the genetic origin of their unique features fascinating researchers for decades.

## Background & Summary

**Fig. 1:**
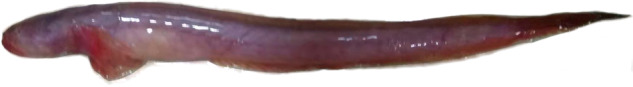
Eel goby, *O. rebecca* collected from the intertidal zone of Zhangzhou, Fujian Province, China.

Gobies have evolved many distinctive morphological features. For example, their pelvic fins have healed to form a suction cup for clinging to rocks in case they are swept away by rapids^1^. The eel goby (*Odontamblyopus rebecca*) (Fig. 1), which belongs to the genus Odontamblyopus (Gobiidae: Amblyopinae)^2^, is an eel-like benthic burrowing fish that lives mainly in warm waters such as the South China Sea and the Indo-West Pacific^3^. As a new component of gobies named in 2003, *O. rebecca* has evolved even more fascinating features that include a unique eel-like body plan, particularly degenerated eyes, and a benthic lifestyle. In recent years, studies of *O. rebecca* have mainly focused on geographic distribution patterns^4,5^ and phylogeny based on mitochondrial genome data^6^. However, the unique phenotypic characteristics of *O. rebecca* and its molecular mechanism cannot be fully understood by analyzing the mitochondrial genome data alone. Therefore, a high-quality genome assembly to obtain an accurate annotation of the protein-coding genes as a basis for a full understanding of the genetic mechanism of the unique phenotypes is particularly important for *O. rebecca*.

In recent years, the rapid development of high-throughput sequencing technology and their gradual reduction in costs has made large-scale genome sequencing and assembly feasible in non-model taxa. Among them, next-generation sequencing (NGS) is highly accurate but is limited to short-read-length (typically 100 bp or 150 bp) sequencing and is thus not ideal for handling repetitive sequences. Meanwhile, third-generation sequencing (TGS) takes the advantage of long-read-length (typically 20–30 Kb) sequencing, but compromise in sequencing accuracy at single-base level^7^. Therefore, the prevailing genome-assembly strategy is to incorporate the merits of both sequencing technology by assembling the reference genome using TGS data while correcting assembly errors using NGS data. In combination with high-throughput chromosome conformation capture technology (Hi-C), the genome can be further assembled to the chromosome-level^8^. Such genome assembly strategy has been employed to address many important scientific problems in teleost fishes, to date^9–11^.

In this study, we used next-generation DNBSEQ short reads (MGI Tech Co., Ltd, Shenzhen, China), third-generation Nanopore long reads (Oxford Nanopore Technologies (ONT)), Hi-C and RNA-Seq sequencing data to assemble and annotate the *O. rebecca* genome. The results revealed an assembled genome size of 918.57 Mb with 23 pseudochromosomes anchored. The completeness of the genome assembly was assessed using a number of parameters, which include scaffold N50 score (41.67 Mbp), BUSCO score (97.75%), mapping ratio of short reads (99.65%) and transcripts (99.82%), indicating the high contiguity and quality of the genome assembly. In addition, 22,999 protein-coding genes were successfully predicted, of which 21,855 gene were functionally annotated in the public database, indicating the reliability of our predictions. The assembled chromosome-level genome of *O. rebecca* would not only provide important genomic resources for phylogenetic and comparative genomic studies of eel gobies, but also expand our understanding on the possible genetic origin of their unique features such as eel-like body plan, particular degenerated eyes fascinating researchers for decades.

## Methods

### Sampling, library construction, and sequencing

The *O. rebecca* sample was collected from the intertidal zone of Zhangzhou, Fujian Province, China. Briefly, dissection was performed in a sterilized environment, and organs including muscle, liver, and intestine were sampled and snap-frozen in liquid nitrogen for nucleic acid extraction. All anatomical procedures comply with relevant ethical regulations provided by the Institutional Animal Care and Use Committee of Zhejiang Ocean University, Zhejiang, China (Protocol Number: 2023082). Genomic DNA was extracted from muscle using the QIAGEN kit (QIAGEN, Cat. No. 13343). The total RNA was extracted from muscle, liver, and intestine using TRIzol reagent (Invitrogen, Carlsbad, CA, USA)^12^. After extraction, the size and integrity of the extracted DNA and RNA were evaluated using 1% agarose gel electrophoresis, and the concentration and purity of DNA and RNA were further analyzed using a Nanodrop 2000c ultraviolet spectrophotometer. For genome assembly of *O. rebecca*, Nanopore sequencing libraries were first prepared with the SQK-LSK109 Ligation Sequencing Kit (Oxford Nanopore Technologies) following the manufacturer’s instruction. The prepared libraries were sequenced on R9.4.1 flow cells using a PromethION DNA sequencer (Oxford Nanopore Technologies) platform to generate the Nanopore long reads data. Secondly, short-insert (350–700 bp) paired-end libraries were constructed using the MGIEasy FS DNA Library Prep Kit (BGI, Cat. No.1000006988) and sequenced on the MGIDNB (MGIDNB T7) platform to generate the DNBSEQ short reads data to correct and evaluate the assembly from the extracted genomic DNA of *O. rebecca*. In addition, the Hi-C libraries were also constructed to generate Hi-C data to obtain chromosome-level genome assemblies using the isolated genomic DNA after fragmented and purified using magnetic beads. For genome annotation of *O. rebecca*, the complementary DNA libraries were constructed from RNA isolated from muscle, liver, and intestine using VAHTS Universal V6 RNA-seq Library Prep Kit for MGI (Vazyme, NRM604) according to the manufacturer’s instructions. For this purpose, the oligo dT magnetic beads were used to capture the mRNA, and then interrupted with the magnesium ions. The interrupted mRNA is reverse transcribed into a short cDNA using random primers, and end repair and A-tail addition were performed and sequenced also on the MGIDNB platform.

### Quality control of raw sequencing data

**Fig. 2:**
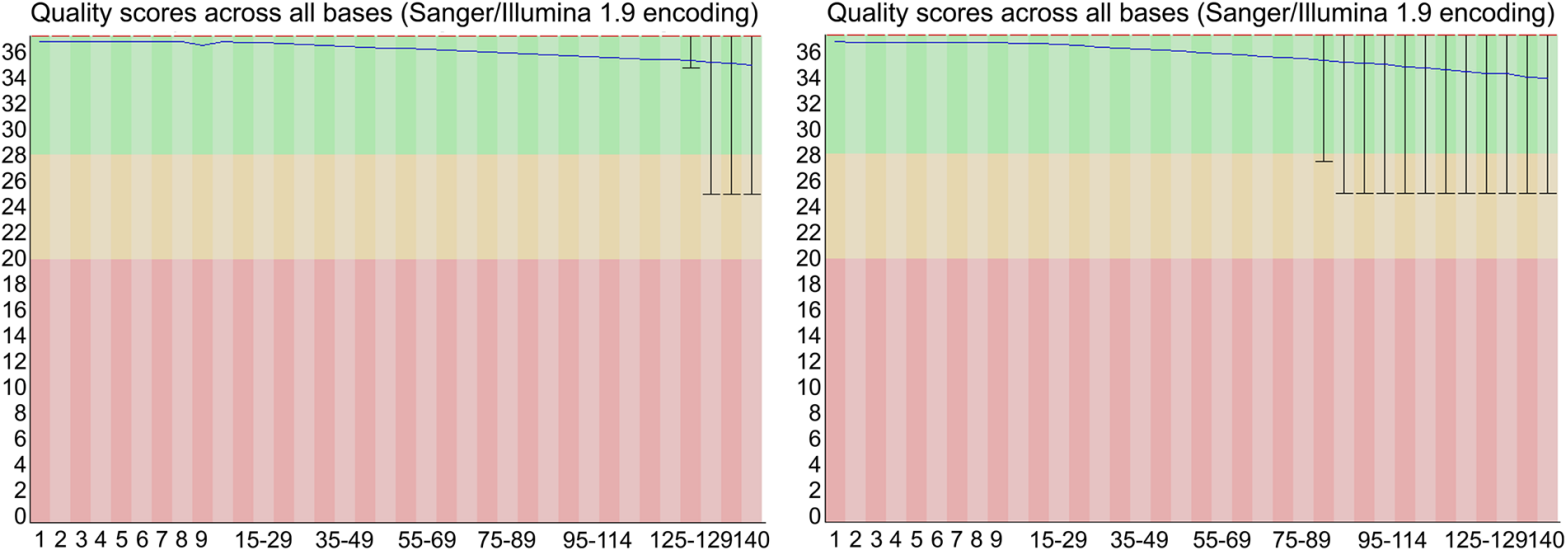
Base quality examined for DNBSEQ short reads using FastQC software. X-axis represents the position in reads, and the Y-axis represents the quality scores across the bases. The colored area illuminates the low (pink), middle (yellow) and high (green) quality scores of the bases.

All raw sequencing data generated in this study were filtered to remove adaptors, low-quality bases, and duplicate reads using different strategies depending on the platform used. For the DNBSEQ short reads, we used fastp software v0.23.2^13^ to remove adaptor sequences, low-quality reads, and short sequences with parameters set as “-l = 50, -w = 6”. Then, we checked the quality of the cleaned data using FastQC software v0.11.9^14^ and found very high base scores in these data, indicating the high-quality of the sequencing data we obtained (Fig. 2). For the Nanopore long reads, the reads were filtered using the NanoFilt software v2.8.0^15^ with the parameter of “-q = 7”. The Hi-C data and RNA-seq data were filtered using the same method and parameter settings as for the DNBSEQ short reads. Finally, we obtained 48.21 Gbp of DNBSEQ short reads (Table 1), 84.64 Gbp of Nanopore long reads with an N50 length of 27.72 Kb (Table 2), and 146.02 Gbp of Hi-C sequencing data (Table 3). In addition, we obtained 41.10 Gbp of liver transcriptome data, 15.78 Gbp of muscle transcriptome data, and 6.62 Gbp of intestine transcriptome data (Table 4).

**Table 1:** Statistics of the genome sequencing data generated from MGIDNB T7 platform.

Library name	DNBSEQ reads number	DNBSEQ reads base (bp)	Sequencing strategy
D2113807A_L2_344 × 44	160,714,440	48,214,332,000	PE150

**Table 2:** Statistics of the sequencing reads generated from Nanopore platform.

ONT reads number	ONT reads base (bp)	Max ONT reads length (bp)	Average ONT reads length (bp)	ONT reads N50 (bp)
5,062,627	84,638,741,712	186,028	16,718	27,720

**Table 3:** Statistics of the Hi-C sequencing data generated from MGIDNB T7 platform.

Library name	Raw reads number	Raw Bases (bp)	Sequencing strategy
**HIC989**	486,728,315	146,018,494,500	PE150

**Table 4:** Statistics of RNA-seq data generated from MGIDNB T7 platform.

Read_number	Len_all(bp)	Source
136,986,393	41,095,917,900	Liver
52,594,601	15,778,380,300	Muscle
22,076,625	6,622,987,500	Intestines
211,657,619	63,497,285,700	Total

### Genome size estimation

**Fig. 3:**
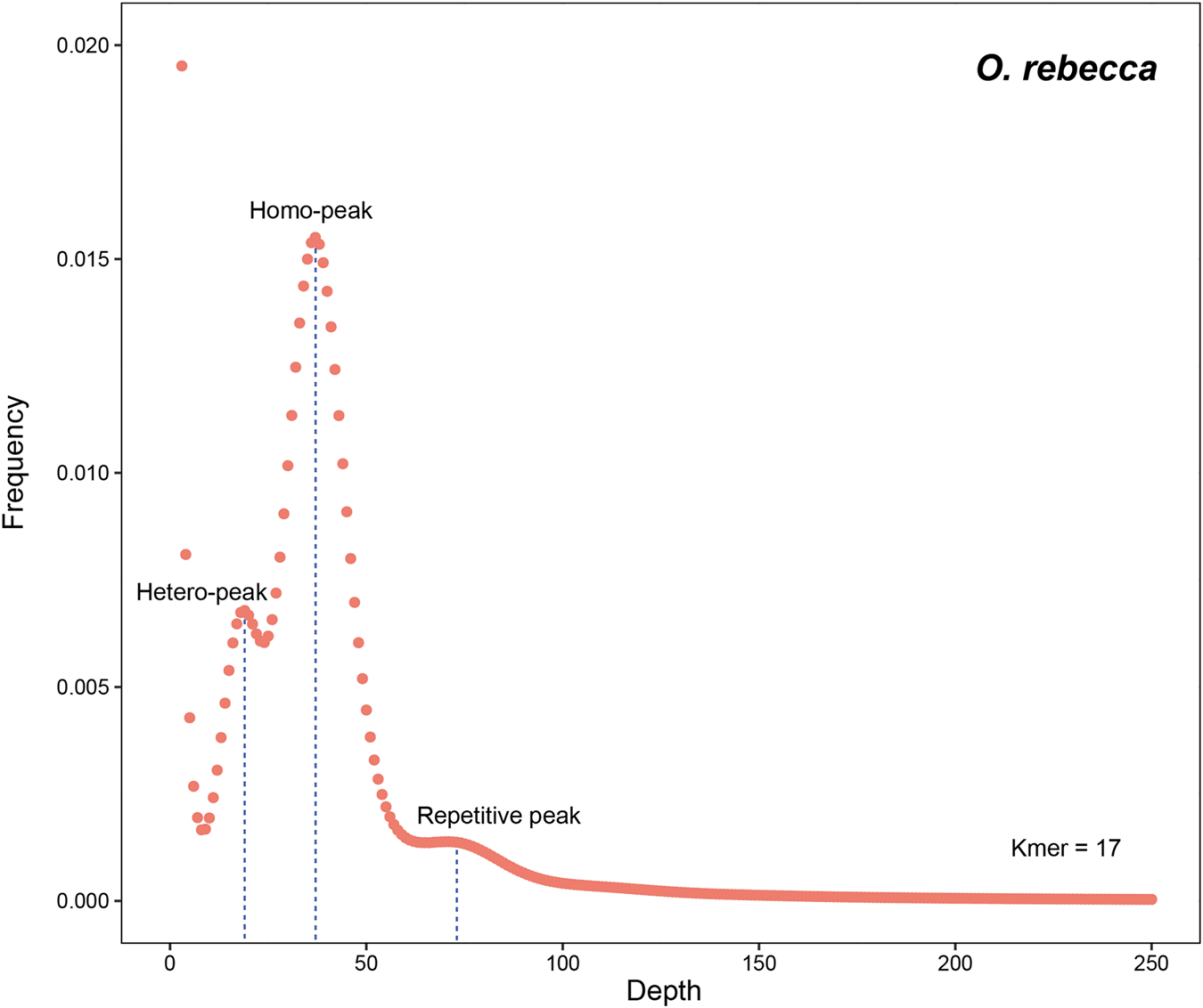
K-mer analysis for the genome size evaluation in *O. rebecca*. The distribution of 17-mer frequency in *O. rebecca* genome was shown. The X-axis represents the k-mer depth, and the Y-axis represents the frequency of the k-mer for a given depth. The first, second and third peaks in the figure corresponded to the heterozygous homozygous, and repeated Kmers, respectively.

DNBSEQ short reads were used to estimate the genome size based on k-mer analyses. To this end, all filtered high-quality DNBSEQ short reads data were calculated using kmerfreq v1.0^16^ with the parameters of “-k = 17, -l”. Here, the 17-mer was selected because such k-mer size was demonstrated capable of generating adequate unique k-mer sequences for a sound genome size evaluation when the genome size falls into a scope of what is typical in Gobiidae^17–19^. The genome size was estimated using the formula: genome size = TKN_17-mer_/PKFD_17-mer_, where TKN_17-mer_ is the total number of k-mers and PKFD_17-mer_ is the peak frequency depth of the 17-mer. The estimated genome size was then used to evaluate the subsequent result of the genome assembly. The results revealed an estimated genome size of ~903.03 Mbp in *O. rebecca*. The kmer distribution of the genome consists of three peaks (Fig. 3), which may correspond to the heterozygous, homozygous, and repeated k-mers, respectively, as usually observed in many other teleost fishes^20,21^.

### Genome assembly

**Fig. 4:**
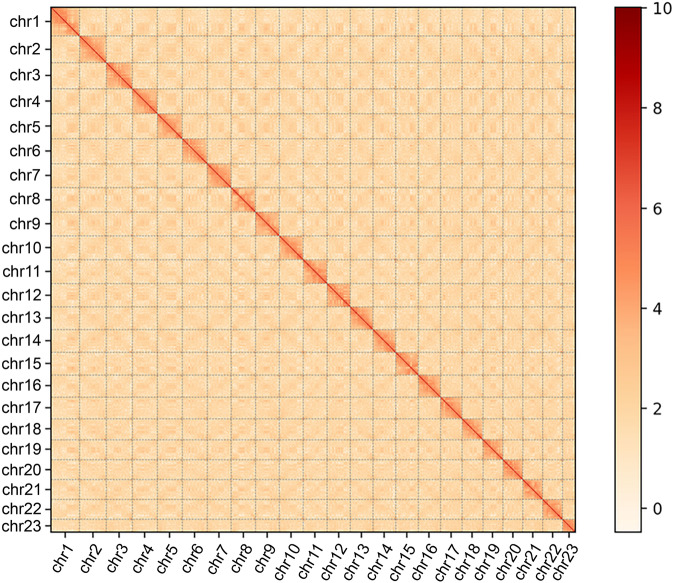
A heatmap of chromosome interaction in *O. rebecca*. The blocks represent the 23 pseudochromosomes. The color bar illuminates the contact density from white (low) to red (high).

**Fig. 5:**
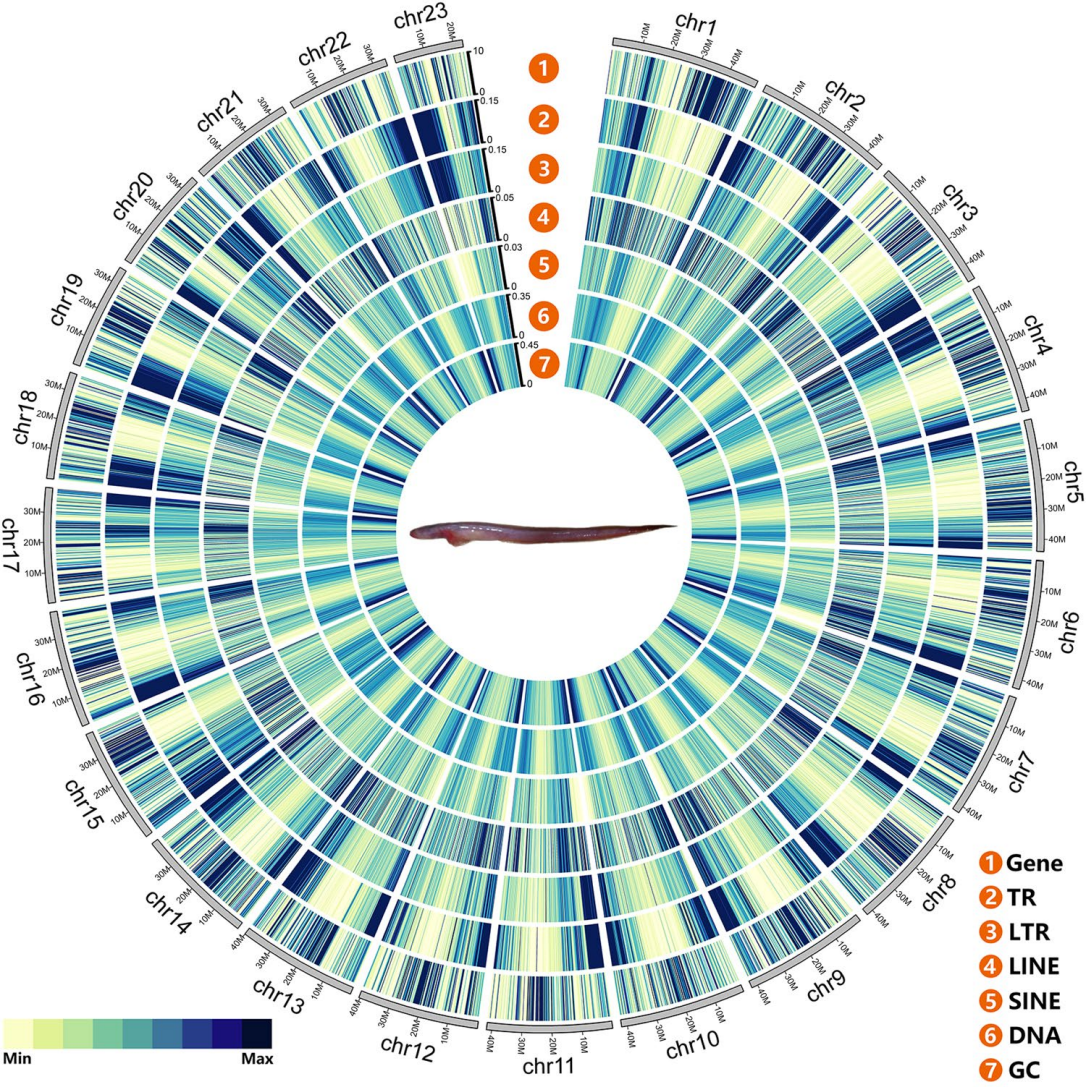
Circos plot of the distribution of the genomic elements in *O. rebecca*. From outside to inside are the distributions of protein-coding genes, tandem repeats (TRs), long tandem repeats (LTRs), long interspersed nuclear elements (LINEs), short interspersed nuclear elements (SINEs), DNA elements, and GC content in the genome. The color bar illuminates the number/percent of each genomic element in the genome from light (low) to dark (high).

Nanopore long reads have a relatively higher error rate at the single-base level compared to DNBSEQ short reads. Therefore, we first performed error correction on the raw sequencing data, and the resulting Nanopore long clean reads were thereafter assembled into the genome using NextDenovo software v2.4.0 (https://github.com/Nextomics/Next Denovo) with parameters set as “read_type = ont, read_cutoff = 1k, and pa_correction = 3”. To this end, the filtered Nanopore clean data were split and compared with each other using Minimap2 software v2.9^22^ to find overlap areas between reads and remove redundant overlap areas. The string graph algorithm was then applied to assemble high-quality genomes. NextPolish software v1.4.1^23^ was further employed to correct the base errors (SNV/Indel) to improve the accuracy of the genome assembly using the DNBSEQ short reads with the parameters set as “sgs_options = -max_depth 100-bwa, lgs_options = -min_read_len 1k -max_depth 100, lgs_minimap2_options = -x map -ont”. The redundant heterozygous contigs were identified and removed based on sequence similarity and the proportion of redundant parts in total contig length calculated by the Purge_haplotigs software v1.0.4^24^. The preliminary assembly yielded a genome size of 918.80 Mbp with 191 contigs and a contig N50 of 24.75 Mbp (Table 5). Hi-C sequencing data were further used for chromosome assembly by using 3D denovo assembly software v170123^25^ with parameters set as “rounds = 0, stage = polish”. Juicer software^26^ and JuiceBox software v1.11.08^27^ were then used for interaction map generation and error correction (Fig. 4). Finally, 23 chromosomes were obtained with a scaffold N50 of 41.67 Mb (Table 5; Figs. 4, 5), and the assembly rate of contigs into chromosomes was up to 99.96% (Table 6). Such a chromosomes number was consistent with what was observed in other closely related species of *Boleophthalmus pectinirostris, Periophthalmus modestus* (Gobiidae: Oxudercinae) and *Taenioides* sp (Gobiidae: Amblyopina). In addition, all the 23 pseudochromosomes could be distinguished easily based on the heatmap (Fig. 4), and the interaction signal around the diagonal was considerably strong, indicating the high-quality of this genome assembly.

**Table 5:** Statistics of the assembled genome based on the Nanopore and Hi-C data.

Term	Nanopore contigs		Hi-C scaffolds	
	Size (bp)	Number	Size (bp)	Number
N90	3,470,437	54	34,796,486	21
N80	5,431,814	34	35,870,458	18
N70	16,585,930	24	39,107,402	16
N60	22,056,681	19	40,000,674	13
N50	24,749,233	15	41,673,572	11
Max length (bp)	39,439,283		49,748,365	
Total length (bp)	918,808,911		918,570,899	
Total number	191		26	
Number ≥ 10Kbp	191		26	

Note: Nanopore contigs represent the contigs assembled using Nanopore data, and Hi-C scaffolds represent the scaffolds after the chromosome assembly.

**Table 6:** Summary of the chromosome assemblies for *O. Rebecca* based on Hi-C data.

Sequence ID	Sequence Length (bp)	Sequence ID	Sequence Length (bp)
Chr1	49,748,365	Chr13	40,000,674
Chr2	46,373,669	Chr14	39,780,004
Chr3	45,465,555	Chr15	39,118,491
Chr4	44,139,061	Chr16	39,107,402
Chr5	44,036,227	Chr17	38,164,928
Chr6	43,152,584	Chr18	35,870,458
Chr7	42,331,694	Chr19	35,329,946
Chr8	42,208,084	Chr20	35,039,131
Chr9	42,058,667	Chr21	34,796,486
Chr10	41,912,060	Chr22	34,347,949
Chr11	41,673,572	Chr23	23,155,060
Chr12	40,414,056		
Total contig length at chromosomes (bp)		918,224,123	
Total contig length (bp)		918,570,899	
Chromosome/total		99.96%	

### Genome evaluation

The completeness and accuracy of the genome assembly could have been reflected by the statistics of contig/scaffolds N50 analyses (contig: 24.75 Mbp; scaffolds: 41.67 Mbp) as indicated above. Here, the quality of the genome assembly was further assessed using three extra statistics resulting from BUSCO, short reads mapping ratio, and transcripts mapping ratio analyses. (1) For BUSCO analysis, the Benchmarking Universal Single-Copy Orthologs (BUSCO) v5.3.1^28^ software was used to search against a single-copy orthologous gene library of Actinopterygii (https://busco-data.ezlab.org/v5/data/lineages/ actinopterygii_odb10.2021-02-19.tar.gz) to assess the integrity of coding regions from the assembled genome. The results showed that a total of 3,640 core genes were identified, including 3,558 complete genes, 3,517 single-copy genes, 41 multi-copy genes, 30 fragmented genes, and 52 deletion genes, which account for 97.75%, 96.62%, 1.13%, 0.82% and 1.43% of the total genes, respectively (Table 7). (2) For short reads mapping ratio analysis, the genome index was first built by the BWA-MEM software v0.7.17-r1188^29^ using the parameters of “-a bwtsw”. The DNBSEQ short reads were then mapped to the genome to assess the completeness of the assembly. The mapping ratio was calculated by the *flagstat* function of SAMtools software. The results showed that the total mapping rate of DNBSEQ short reads to the genome was 99.65%, the paired mapping rate was 99.64%, and the properly paired mapping rate was 94.74% (Table 8). (3) For transcripts mapping ratio analysis, all the RNA-Seq reads (99.16 Mb) were first assembled into transcripts using StringTie software 1.3.5. Linux_x86_64^30^, and then it was mapped to the genome using BLAT software v37x1^31^. The results showed that a total of 41,624 reads were mapped to the genome, with a mapping rate of 99.82% (Table 9). Taken together, all the results indicated that we had obtained a high-quality chromosome-level assembly of the *O. rebecca* genome.

**Table 7:** Results of the BUSCO assessment for genome assembly in *O. Rebecca*.

Type	Proteins	Percentage (%)
Complete BUSCOs (C)	3,558	97.75
Complete and single-copy BUSCOs (S)	3,517	96.62
Complete and duplicated BUSCOs (D)	41	1.13
Fragmented BUSCOs (F)	30	0.82
Missing BUSCOs (M)	52	1.43
Total BUSCO groups searched	3,640	100.00

**Table 8:** The mapping ratio of the short reads to the assembled genome of *O. rebecca*.

Mapping rate (%)	Paired mapping rate (%)	Properly paired rate (%)
99.65	99.64	94.74%

**Table 9:** The mapping ratio of transcript to the assembled genome of *O. rebecca*.

Mapping rate (%)	Total reads number	Number of alignments
99.82	41,700	41,624

### Annotation of repetitive sequences

To annotate the repetitive elements in the *O. rebecca* genome, including tandem repeats and transposable elements (TEs), we integrated a homology prediction using the Repbase library^32^ (http://www.girinst.org/repbase) and a *de novo* prediction based on self-sequence alignment and repetitive sequence features. The tandem repeat was annotated using Tandem Repeat Finder software v4.09^33^ with parameters were set as “Match = 2, Mismatch = 7, Delta = 7, PM = 80, PI = 10, Minscore = 50, MaxPeriod = 2000 -d -h”. TEs were *de novo* predicted on both DNA and protein levels. On the DNA level, RepeatModeler software v1.0.11^34^ (-database mydb -pa 10) and LTR-FINDER v1.0.7^35^ (-w 2 -o 3 -t 1 -e 1 -m 2 -u -2 -D 20000 -d 1000 -L 3500 -l 100 -p 20 -g 50 -G 2 -T 4 -S 6.00 -M 0.00 -B 0.400 -b 0.400 -O 40 -F 0) were used to build *de novo* repeat library. RepeatMasker software open-4.0.9^36^ (http://repeatmasker.org) (-nolow -no_is -norna -parallel 2) was then run against the *de novo* library and repbase (RepBase v.16.02) separately to identify homologous repeats. On the protein level, RepeatProteinMask v4.0.9 was used to search TEs in its protein database. Finally, the annotation results of all repetitive sequences were merged as a final result. The results showed that a total of 489.68 Mb of sequences were identified as repetitive sequences (including TEs, satellite, simple repeat, others, and unknowns) in the *O. rebecca* genome, accounting for 53.29% of the genome size (Table 10). Among them, 297.90 Mb of transposable elements (TEs) were annotated, accounting for 32.43% of the genome (Table 11). There are four major types of TEs, of which, DNA elements, long interspersed nuclear elements (LINEs), short interspersed nuclear elements (SINEs), and long erminal repeats (LTRs) (Fig. 5) account for 16.12% (148.10 Mbp), 8.49% (78.00 Mbp), 1.07% (9.82 Mbp), and 6.75% (61.98 Mbp) of the genome, respectively.

**Table 10:** Statistics of the annotated repeat sequences in *O. rebecca* genome.

Type	Repeat Size (bp)	% of genome
Trf	105,278,103	11.46
Repeatmasker	140,547,735	15.30
Proteinmask	34,669,414	3.77
De novo	380,331,288	41.39
Total	489,675,645	53.29

**Table 11:** Statistics of the repetitive elements in *O. rebecca* genome.

	RepBase TEs		TE Proteins		De novo		Combined TEs	
	Length (bp)	% in Genome	Length (bp)	% in Genome	Length (bp)	% in Genome	Length (bp)	% in Genome
DNA	65,726,147	7.15	3,774,515	0.41	112,673,397	12.26	148,095,272	16.12
LINE	52,907,281	5.76	26,294,877	2.86	54,090,128	5.89	78,000,619	8.49
SINE	6,434,641	0.70	0	0.00	4,615,307	0.50	9,818,105	1.07
LTR	19,340,283	2.10	4,605,625	0.50	49,248,145	5.36	61,974,924	6.75
Satellite	6,746,526	0.73	0	0.00	3,690,319	0.40	10,355,504	1.13
Simple repeat	0	0.00	0	0.00	828,885	0.09	828,885	0.09
Other	22,121	0.00	0	0.00	0	0.00	22,121	0.00
Unknown	750,022	0.08	0	0.00	164,925,150	17.95	165,656,526	18.03
Total	140,547,735	15.30	34,669,414	3.77	380,331,288	41.39	449,207,484	48.89

### Prediction of protein-coding genes

**Fig. 6:**
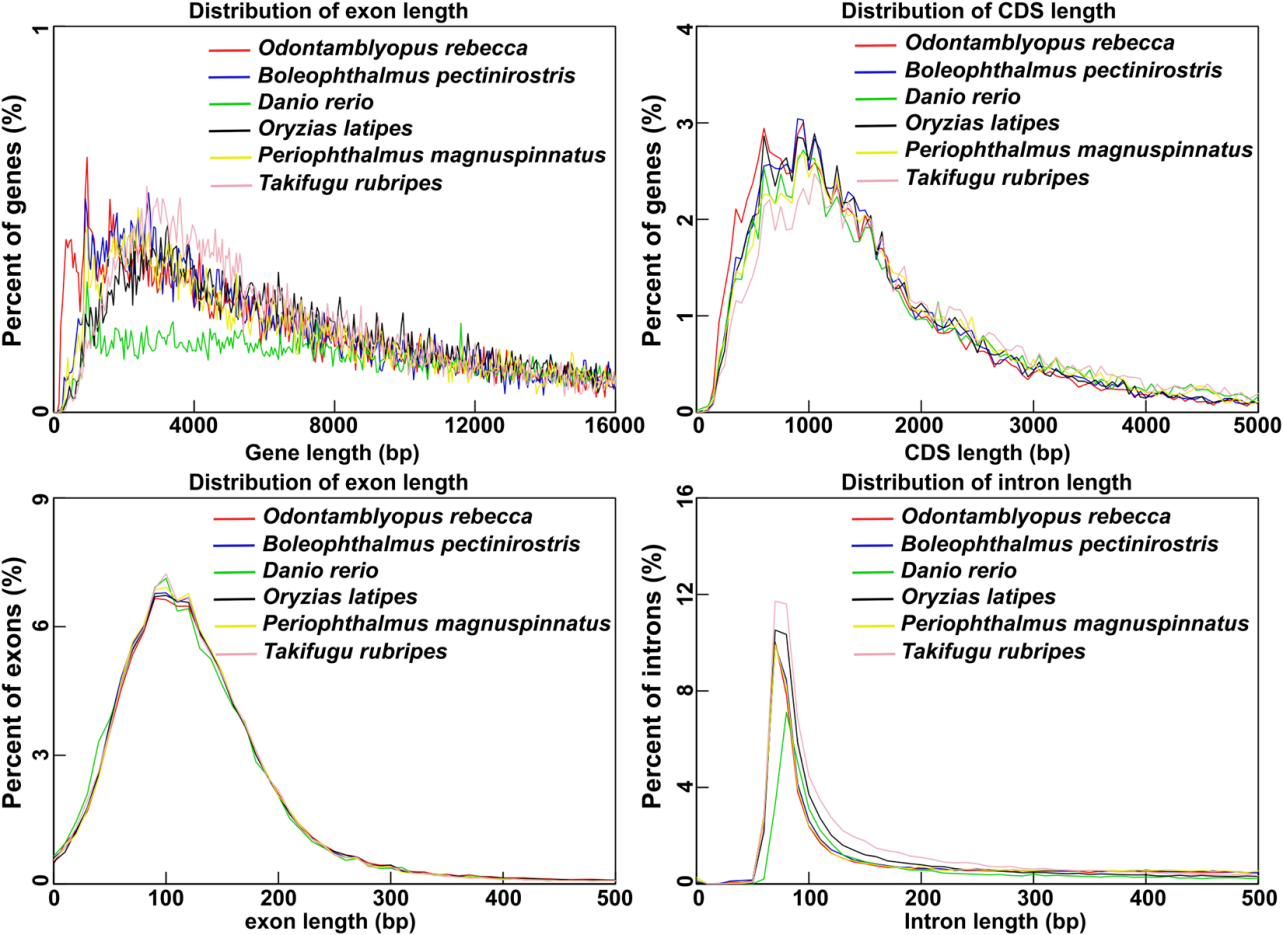
The length of protein-coding genes in *O. rebecca* and their closely related species. The gene length, CDS length, exon length, and intron length were compared among *O. rebecca* and the other five teleost species to verify the quality of gene annotation.

To obtain a high-confidence gene set, a combination of three strategies of *de novo* prediction, homology-based prediction, and transcripts-based prediction were used to annotate the protein-coding genes. (1) For *De novo* prediction, GlimmerHMM software v3.0.4^37^, Genscan software v1.0^38^, and Augustus software v3.3.2^39^ (-species = zebrafish, -uniqueGeneId = true, -noInFrameStop = true, -gff3 = on, -strand = both) were performed. (2) For homology-based prediction, the already predicted protein-coding gene sequences of close-related species, including *Oryzias latipes* (GCF_002234675.1), *Boleophthalmus pectinirostris* (GCF_026225935.1), *Periophthalmus magnuspinnatus* (GCF_009829125.3), *Takifugu rubripes* (GCF_901000725.2) and *Danio rerio* (GCF_000002035.6), were first downloaded from public databases. Then, all sequences were aligned to the *O. rebecca* genome using TBLASTN software v2.11.0+^40^ with an e-value of 0.01. The TBLASTN results were further processed to obtain the final homology-based prediction results for each species with parameters set as “model = protein2genome, showtargetgff = 1” using exonerate software v2.2.0^41^. (3) For transcripts-based prediction, StringTie software v1.3.5 was first used to assemble transcripts with parameters set as “-f 0.1 -m 200 -a 10 -c 2.5 -g 50 -M 1.0”. HISAT2 software v2.1.0^42^ was thereafter used to map the RNA-seq data to the genome with parameters set as “-dta -summary-file -S -x -1 -2”. TransDecoder software v5.5.0 (https://github.com/TransDecoder/TransDecoder) was used to predict the coding region of each transcript with parameters set as “-retain_long_orfs_mode dynamic -retain_long_orfs_length 150 -T 500”. Finally, Maker2 software v2.31.10^43^ was used to integrate the gene annotation results generated by the three methods to obtain the final gene set with parameters set as “-r local -o tmp -p 4”. The results revealed a total of 22,999 protein-coding genes that were successfully predicted in the *O. rebecca* genome (Table 12). We checked the quality of the annotated genes by comparing them with several species that share evolutionary affinity, and the results indicated a similarity in the distributions of mRNA length, CDS length, exon length, and intron length between genomes of *O. rebecca* and those closely related species (Fig. 6), possibly incating they share similar patterns of gene structure distribution as the published genomes.

**Table 12:** Functional annotation of the predicted protein-coding genes in *O. rebecca* genome.

Term	Gene number	Percentage (%)
InterPro	20,437	88.86
GO	15,653	68.06
KEGG_ALL	21,342	92.80
KEGG_KO	14,083	61.23
SwissProt	19,512	84.84
TrEMBL	21,573	93.80
TF	3,577	15.55
Pfam	19,713	85.71
Nr	21,755	94.59
KOG	18,136	78.86
Annotated	21,855	95.03
Unannotated	1,144	4.97
Total	22,999	100.00

### Functional annotation of protein-coding genes

To evaluate the annotation quality and obtain the biological function information of the predicted protein-coding gene set, we compared the protein sequences output in this study with all the existing public protein databases, including InterPro^44^ (2021) (https://www.ebi.ac.uk/interpro/), GO^45^ (5.61–93.0) (http://geneontology.org/docs/go-annotations/), Kyoto Encyclopedia of Genes and Genomes (KEGG)^46^ (3.0) (http://www.genome.jp/kegg/), SwissProt^47^ (2021) (http://www.uniprot.org/), TrEMBL (2021) (http://www.uniprot.org/), TF (AnimalTFDB3.0), Pfam^48^ (01.34.0) (http://pfam.xfam.org), NCBI Non-Redundant Protein Sequence Database (NR) (2021) (https://www.ncbi.nlm.nih.gov/refseq/about/non-redundantproteins/), and Eukaryotic Orthologous Groups of Proteins (KOG) (2003) (ftp://ftp.ncbi.nih.gov/pub/COG/KOG/kyva). Functional information was analyzed using BLAST software v2.31.10^49^. The results showed that a total of 21,855 genes could be annotated, accounting for 95.03% protein-coding genes, and only 1,144 genes could not be annotated, accounting for 4.97% protein-coding genes (Table 12), further suggesting we got a reliable assembly and annotation of *O. rebecca* genome.

## Data Records

The genomic DNBSEQ short-insert sequencing data were deposited in the Sequence Read Archive at NCBI SRR25064244^50^. The genomic Nanopore sequencing data were deposited in the Sequence Read Archive at NCBI SRR25064242^51^. The transcriptome sequencing data were deposited in the Sequence Read Archive at NCBI SRR25064238^52^, SRR25064239^53^, SRR25064240^54^, and SRR25064243^55^. The Hi-C sequencing data were deposited in the Sequence Read Archive at NCBI SRR25064241^56^. The final genome assembly was deposited in GenBank at NCBI with the accession number ASM3068695v1^57^, the Submitted GenBank assembly number is GCA_030686955.1, the BioProject number is PRJNA977196, and the BioSample ID is SAMN35453534. The annotation results of repetitive sequences, gene structure, and functional prediction were deposited in the Figshare database under DOI code: 10.6084/m9.figshare.23689398^58^.

## Technical Validation

### Genome evaluation

The quality of *O. rebecca* genome assembly was evaluated using N50, BUSCO, short reads mapping ratio, and transcripts mapping ratio analyses. Results showed that the assembly contained good contiguity, a high percentage of complete and single-copy genes, had a high mapping rate of short reads and transcripts, indicating a high-quality assembly.

## Data Availability

The software used in this study is in the public domain, with parameters clearly described in Methods. Where detailed parameters were not provided for the software, default parameters were used instead, as suggested by the developers. No custom script or code was used.
